# P-493. Prevalence of Hepatitis B-HIV coinfection among adolescents: a survey of seven Sub-Saharan African countries

**DOI:** 10.1093/ofid/ofaf695.708

**Published:** 2026-01-11

**Authors:** Derrick Abila, Eddy Kyagulanyi, Beliza Chemutai

**Affiliations:** Uganda Child Cancer Foundation, Kampala, Kampala, Uganda; Makerere University College of Health Sciences, Kampala, Kampala, Uganda; Makerere Lung Institute, Kampala, Kampala, Uganda

## Abstract

**Background:**

Hepatitis B-HIV co-infection has been demonstrated to have a poorer prognosis with hepatic-related deaths demonstrated to be up to 19 times in populations with both infections compared to clients living with HIV only. 2.6 million people have been estimated to be living with both hepatitis B and HIV in Africa, with the prevalence among pregnant women estimated to be approximately 3.8% in Sub-Saharan Africa. However, the prevalence of hepatitis B-HIV co-infection and its associated risk factors among adolescents living with HIV in Sub-Saharan Africa is currently unknown which this study explored.

**Methods:**

his was a cross-sectional study where an analysis of data from 2016-2019 population based health surveys in seven Sub-Saharan African countries was done. Each country’s data was cleaned separately and combined for analysis using STATA 18. Adolescents living with HIV aged 10-19 years were included. Variables that measured demographics, viral load suppression, CD4 count, Hepatitis B status of both the adolescents and their mothers were included. CD4 count was categorised into a binary variable i.e. below 500 cells/mm3 and 500 cells/mm3 or more. Chi square test was done with p value of < 0.05 used as a measure of statistical significance.

**Results:**

Data from 409 adolescents living with HIV was collected, of which 314 (76.8%) were aged between 15-19 years, majority (284 adolescents or 69.4 %) being female. Only 49.6% (198 adolescents) were HIV suppressed with 44.4% (116 adolescents) having a CD4 count of < 500 cells/mm3. The prevalence of Hepatitis B-HIV coinfection was 4.6% (19 adolescents) with the mother’s positive hepatitis B status being the only significant associating factor (p < 0.001).

**Conclusion:**

Our findings highlight the need for active screening of hepatitis B among adolescents living with HIV, with integration of hepatitis B preventive and treatment services to routine care of adolescents living with HIV. In addition, vertical transmission was shown to be the commonest mode of hepatitis B transmission among the adolescents living with HIV which calls for further strengthening of the measures that prevent hepatitis B transmission from mother to child.

**Disclosures:**

All Authors: No reported disclosures

